# The Effects of Delivery Mode on the Gut Microbiota and Health: State of Art

**DOI:** 10.3389/fmicb.2021.724449

**Published:** 2021-12-23

**Authors:** Chenchen Zhang, Lixiang Li, Biying Jin, Xinyan Xu, Xiuli Zuo, Yanqing Li, Zhen Li

**Affiliations:** ^1^Department of Gastroenterology, Qilu Hospital, Cheeloo College of Medicine, Shandong University, Jinan, China; ^2^Laboratory of Translational Gastroenterology, Qilu Hospital, Cheeloo College of Medicine, Shandong University, Jinan, China; ^3^Robot Engineering Laboratory for Precise Diagnosis and Therapy of GI Tumor, Qilu Hospital, Cheeloo College of Medicine, Shandong University, Jinan, China

**Keywords:** cesarean section, vaginal delivery, gut microbiota, intestinal epithelial cells, immune system, probiotics

## Abstract

The delivery mode is an important factor driving alteration in the gut microbiota during the neonatal period. Several studies prove that the alteration of gut microbiota induced by cesarean section could influence the activation of intestinal epithelial cells and the development of immune system. Further, some autoimmune and metabolic disorders may be related to the microbiota dysbiosis in infants caused by cesarean section. It is noteworthy that probiotics could promote the intestinal microecology, which may further prevent and treat cesarean section related diseases. This review summarized the great significance of delivery mode on microbiota and health, as well as provided clinically feasible methods for the prevention and treatment of cesarean section related gut diseases.

## Introduction

A major determinant of the composition of neonatal microbiota is the delivery mode (Penders et al., 2006). Cesarean section (c-section) is an important operation in the field of obstetrics, which is a lifesaving intervention to avoid the risk of mother and child (Sandall et al., 2018). However, in recent years, c-section has been overused. The proportion of c-section delivered infants has increased year by year, especially in China, which is close to 50%. The differences of delivery mode have been linked with the differences of gut microflora of infants (Dominguez-Bello et al., 2010). The birth canal contains probiotics such as *Lactobacillus reuteri*, *L. rhamnosus* and so on. Compared with the c-section infants, the oral cavity, nasal cavity, skin and other parts of vaginally delivered infants will be exposed to more beneficial bacteria. The bacterial communities in vaginally delivered infants are similar to those of the maternal vagine. Studies have confirmed that the gut microbiota of c-section infants is significantly different from that of vaginally delivered infants. The bacterial communities in c-section infants are similar to those found on their own mother’s skin surface (Goedert, 2016). The mode of delivery may give rise to variations in the microbial development, which may then result in alterations in normal physiology or disease susceptibility (Dominguez-Bello et al., 2010). It was also found that the vaginal microbial transmission is conducive to the establishment of the initial gut microbial structure of neonates (Dominguez-Bello et al., 2010, 2016; Shao et al., 2019). In this review, we summarize recent studies concerned that c-section is closely related to an increased risk of food allergy, asthma, diabetes, obesity and other autoimmune and metabolic diseases in children. We also try to explain the possible mechanism that the changes of gut microbiota induced by c-section influence the activation of intestinal epithelial cells and the development of immune system. Probiotics can improve the development of gut microbiota, intestinal barrier and immune system in infants, and may also be used to prevent and treat c-section related gut diseases. These findings contribute to understand the influences of the delivery mode on diseases *via* gut microbiota.

## Influences of Delivery Mode on the Gut Microbiota

Human gut is the habitat of diverse and dynamic microbial ecosystems. Gut microbiota is involved in many basic metabolic pathways and the maturation of the immune system in host. The composition of gut microbiota is unique to each individual after bacterial colonization in infants, though more than 95% of gut microbiota can be divided into four major phyla (Landman and Quévrain, 2016). The establishment of gut microbiota during infancy may be affected by many factors, including the delivery mode (Rutayisire et al., 2016). A number of studies have correlated delivery mode with significant variations of gut microbiota in infants (Table 1).

**TABLE 1:** Studies on the association between the delivery mode and gut microbiota.

	Author	Year	Countries	Study design	Method	Predominant gut microbiota in vaginally delivery infants	Predominant gut microbiota in cesarean section infants	References
1	Shao et al.	2019	United Kingdom	n1^1^ = 314, n2^2^ = 282. During their neonatal period (≤1 month), fecal samples are collected from all babies at least once. During infancy (8.75 ± 1.98 months), 302 babies are resampled later. Maternal fecal samples are obtained from 175 mothers paired with 178 babies.	Longitudinal sampling and whole-genome shotgun metagenomic analysis.	*Bifidobacterium* (such as *Bifidobacterium breve* and *Bifidobacterium longum*), *Bacteroides* (*Bacteroides vulgatus*), *Escherichia* (*Escherichia coli*) and *Parabacteroides* (*Parabacteroides distasonis*).	*Enterococcus faecium*, *Enterococcus faecalis*, *Streptococcus parasanguinis*, *Staphylococcus epidermis*, *Klebsiella pneumoniae*, *Klebsiella oxytoca*, *Clostridium perfringens*, and *Enterobacter cloacae*. Opportunistic pathogens associated with the hospital environment (including *Klebsiella* species, *Enterobacter* and *Enterococcus*).	Shao et al., 2019
2	Dominguez-Bello et al.	2010	Venezuela	n1^1^ = 4, n2^2^ = 6. Before delivery, maternal skin, oral mucosa, and vagina are sampled 1 h. After delivery, neonates’ skin, nasopharyngeal aspirate, and oral mucosa are sampled <5 min, and meconium <24 h.	Multiplexed 16S rRNA gene pyrosequencing.	*Lactobacillus*, *Prevotella*, and *Sneathia* spp.	*Staphylococcus*, *Corynebacterium*, and *Propionibacterium* spp.	Dominguez-Bello et al., 2010
3	Dominguez-Bello et al.	2016	United States	n1^1^ = 7, n2^2^ = 11. During the first two minutes of birth, these neonates delivered by c-section are exposed to their maternal vaginal contents by wiping with gauze, beginning with the mouth, next the face, and lastly the rest of the body. A total number of 1,519 samples are obtained from the oral, skin and anal of infants and mothers. These samples are obtained at six time points (1, 3, 7, 14, 21, and 30 days) during the first month of life after birth.	Sequencing the V4 region of 16S rRNA gene.	*Lactobacillus* are enriched in the early stage followed by a bloom of *Bacteroides* from the second week.	There is no such phenomenon.	Dominguez-Bello et al., 2016
4	Akagawa et al.	2019	Japan	Fecal samples of 36 healthy Japanese neonates are obtained at 4 days and 1 month of age.	16S rRNA sequencing	*Bacteroidales* and *Enterobacteriales.*	*Bacillales* and *Lactobacillales.*	Akagawa et al., 2019

*^1^n1 = the number of vaginally delivered infants;*

*^2^n2 = the number of cesarean section infants.*

Fecal samples were collected from 596 healthy, full-term infants, including 282 c-section delivered infants and 314 vaginally delivered infants (Shao et al., 2019). During neonatal period (≤1 month), fecal samples of these babies are collected at least once. Fecal samples are resampled later from 302 babies during infancy (8.75 ± 1.98 months). Besides, they also collect the fecal samples from 175 mothers paired with 178 babies. Metagenomic analysis of the total 1,679 fecal samples reveals that the delivery mode is the most important factor leading to the variation of gut microbiota during neonatal period. On the fourth day, the largest influence of the delivery mode is observed (R2 = 7.64%, *P* < 0.001). Some studies suggest that early-life antibiotic exposures and breastfeeding play an important role in the establishment of gut microbiota (Bokulich et al., 2016; Kim et al., 2019), however, in Shao’s work, some meaningful clinical covariates which are related to hospital delivery (such as the duration of hospitalization and the perinatal antibiotic usage), as well as breastfeeding, exhibit smaller effects. The influence of delivery mode decreases with age but remains prominent when sampling during infancy (R2 = 1.00%, *P* = 0.002). Maternal-infant transmission of gut microbiota is an important mode of bacterial transmission. They profile the gut microbiota across 178 mother–baby dyads in order to evaluate whether the changes of neonatal microbiota are related to the differential transmission of maternal microbiota. The results indicated that 74.39% of the early microbiota in vaginally delivered infants is obtained from maternal microbial strains. In contrast, only 12.56% of the early microbiota in c-section delivered infants is obtained from maternal microbial strains. *Parabacteroides* spp., *Bacteroides* spp., *Bifidobacterium* spp., and *E. coli* are most commonly transmitted to infants from mothers *via* vaginal delivery. Most of c-section delivered infants still cannot acquire *Bacteroides* species, such as *B. vulgatus*, after neonatal period. These results emphasize that neonatal period is a crucial early window for transmission of gut microbiota from mother to infant. Dominguez-Bello et al. (2010) have found that the composition of gut bacterial communities in vaginally delivered infants is similar to that of the maternal vaginal microbiota, dominated by *Prevotella*, *Sneathia*, and *Lactobacillus* spp., while that of c-section delivered infants is similar to that of mothers’ skin surface, dominated by *Propionibacterium*, *Corynebacterium*, and *Staphylococcus* spp. The maternal vaginal microbiota provides the physical habitats where the first natural microbial exposure to neonates. Differences in microbial succession modes of body habitats, such as the gut, may be caused by differences in initial communities, which will continue over time. The *Lactobacillus* are enriched in the gut at the early stage of vaginally delivered neonates, and then *Bacteroides* increased in the second week. However, c-section delivered infants do not have these above microbial features (Dominguez-Bello et al., 2016). In 2019, Akagawa et al. (2019) demonstrated that at 4 days of age, the bacterial diversity of vaginally delivered neonates was significantly higher than those delivered by c-section. *Enterobacteriales* and *Bacteroidales* in vaginally delivered neonates are overrepresented (*P* = 0.011 and *P* = 0.0031), while *Lactobacillales* and *Bacillales* in c-section delivered neonates are overrepresented (*P* = 0.0016 and *P* = 0.012). However, there is little difference in relative abundance and bacterial diversity among infants at 1 month of age. It was considered that c-section delivery appeared to decrease the diversity of gut microbiota in neonates, leading to dysbiosis. However, since breast milk may help correct the dysbiosis induced by c-section, this situation improves to the equivalent level seen in 1-month-old vaginally delivered infants. It was found that transplant the maternal vaginal microbiota to their children delivered by c-section could partially restore the above-mentioned microbiota characteristics, although the long-term effect of the transplantation had yet to be further confirmed by long-term observation (Dominguez-Bello et al., 2016). These studies indicate that delivery mode is a significant factor affecting the gut microbiota of infants.

Rutayisire et al. (2016) found that the colonization pattern and diversity of gut microbiota were obviously correlated with the delivery mode in the first three months of life, but the significant differences observed disappeared after 6 months of life. Meanwhile, Bokulich et al. (2016) showed that, compared to vaginally delivered infants, c-section delivered infants exhibited significantly decreased evenness, richness, and phylogenetic diversity at baseline during the first month of life. Subsequently, children delivered by c-section showed lower richness and diversity up to the age of 2, especially after 8 months of age. However, other studies suggested that microbiota dysbiosis may persist much longer, until the age of 2 or even 7 (Salminen et al., 2004; Jakobsson et al., 2013). Different confounding and concurrent factors can partly explain the differences in the results obtained by different authors. These factors are not always correctly identified as neonatal exposure (such as feeding type), or fully reported (such as geographical or ethnic differences), or even due to factors associated with the experimental technology used (DNA extraction method, culture technology, etc.) (Arboleya et al., 2018).

## Long-Term Health Consequences of Gut Microbial Dysbiosis Induced by C-Section

The gut of human contains approximately 10^14^ bacteria and many other microorganisms (Landman and Quévrain, 2016). The symbiotic relationship between host and gut microbiota ensures proper development of the metabolic system in humans (Patterson et al., 2016). A variety of perinatal determinants, such as c-section delivery, could influence the pattern of bacterial colonization and lead to dysbiosis (Butel et al., 2018). Dysbiosis is usually driven by a set of host-related and environmental factors, which contribute to alterations in the composition and function of the gut microbiota (Levy et al., 2017). Dysbiosis is characterized by the expansion of potentially harmful microbiota, the loss of beneficial microbiota, and/or the loss of overall microbial diversity (Petersen and Round, 2014). Long-term dysbiosis of gut microbiota may have long-lasting functional effects and result in a variety of diseases. The alterations of the microbiota are important causes of increased risk of food allergy, asthma, diabetes and obesity.

### Microbiota and Food Allergy

In recent decades, the prevalence of food allergy has been rising with economic growth and urbanization, which affects up to 10% of populations all over the world (Tang and Mullins, 2017; Sicherer and Sampson, 2018). The increased incidence of allergic diseases is related to changes in environmental factors and human lifestyle, including changes in external and internal microbial communities. C-section delivery may be susceptible to allergic disorders, which presumably due to the changes in the establishment of normal gut microbiota during early infancy (Papathoma et al., 2016; Mitselou et al., 2018). Recent evidence suggests that the increase in prevalence of food allergy is related to alterations in the gut microbial composition and function (Azad et al., 2015; Chen et al., 2016).

In 2016, Chen et al. (2016) conducted a case-control study on 22 healthy children and 23 children with food allergy. It was found that food allergy was related to the alterations of gut microbiota. There is lower diversity of the total microbiota (*P* = 0.01) in children with food allergy. Compared with healthy children, the number of Proteobacteria, Actinobacteria, and Firmicutes are obviously increased at the phylum level and that of *Bacteroidetes* bacteria is obviously reduced in children with food allergy. Besides, significant differences are observed in the children with food allergy at the genus level, including the elevated abundances of *Subdoligranulum* and *Clostridium* IV, and the depressed abundances of *Veillonella* and *Bacteroides*. In 2015, Azad et al. (2015) performed a general population cohort study of 166 infants and found that gut colonization in infancy might increase the risk of developing food allergy and atopic disease. The low abundance of gut microbiota and the elevated ratio of Enterobacteriaceae to Bacteroidaceae in early infancy are related to subsequent food allergy. There is a 55% decrease in the risk of food allergy at 1 year when the abundance of microbiota in infants increases by every quartile at 3 months (aOR 0.45, 95% CI: 0.23–0.87). Separately, a two-fold increase in risk is related to each quartile increase in the proportion of Enterobacteriaceae to Bacteroidaceae (2.02, 1.07–3.80). At 1 year, the proportion of Enterobacteriaceae to Bacteroidaceae remains elevated in infants with food allergy, while the abundance of Ruminococcaceae tends to decrease. These results suggest that the dysbiosis of gut microbiota is linked to an elevated risk of allergic diseases.

### Microbiota and Asthma

Asthma is a common chronic and non-communicable diseases in children (Papi et al., 2018), which can be characterized by intermittent respiratory symptoms, reversible airflow obstruction and airway inflammation (Levy and Fleming, 2020). Asthma is thought to be a result of the complex interaction between environment and gene. Recent studies have proved c-section is associated with asthma (Moya-Pérez et al., 2017; Stokholm et al., 2020). As described before, c-section has a great impact on the earliest microbiota and its development (Dominguez-Bello et al., 2010, 2016; Shao et al., 2019). Studies have also found that early-life dysbiosis of microbiota is an important factor affecting the development of asthma (Table 2; Holgate, 2012; Arrieta et al., 2015; Fujimura et al., 2016; Stokholm et al., 2018).

**TABLE 2:** Studies on the association between the gut microbiota and asthma.

	Author	Year	Countries	Study design	Method	Analysis of the relationship between gut microbiota and the risk of asthma	References
1	Arrieta et al.	2015	Canada	Cohort of 319 infants. According to allergy skin prick testing (that is, atopy) and clinical wheeze data at the age of 1, these infants were divided into 4 different clinical phenotypes: controls (*n* = 74), atopy only (*n* = 87), wheeze only (*n* = 136), and atopy + wheeze (AW, *n* = 22).	16S rRNA gene sequencing, quantitative PCR and PICRUSt analysis, fecal SCFAs and urinary metabolomic analysis.	During the first 100 days of life, infants at risk of asthma show gut microbial dysbiosis transiently, which is characterized by the decrease of four bacterial genera-*Rothia*, *Veillonella*, *Faecalibacterium* and *Lachnospira*, and the increased risk of asthma. Children in the AW phenotype present this gut microbial characteristic at 3 months, and compared with the control group, the AW group at the age of 3 are 21.5 times more likely to develop asthma. Atopic dermatitis and antibiotic exposure in the first year of life were factors that increase infants’ risk of being classified as AW compared controls.	Arrieta et al., 2015
2	Fujimura et al.	2016	Southeastern Michigan	Cohort of 298 infants. Assuming that there exist human neonatal intestinal microbiota (NGM) with different composition, which are associated with relative risk (RR) of childhood asthma. Newborn (median age, 35 days) were divided into three microbiota composition states (NGF1-3) according to fecal samples (age 1–11 months) and 16S rRNA sequencing.	16S rRNA sequencing, Bacterial- and fungal-community profiling, PICRUSt and metabolomic analyses.	Neonatal gut microbiota affect the susceptibility of allergic asthma in children, possibly affecting CD4 + T cell populations and function through changes in the gut microenvironment. Labeled NGM3, the highest risk group, exhibit higher relative abundance of particular fungi (for example, *Rhodotorula* and *Candida*), lower relative abundance of certain bacteria (*Faecalibacterium*, *Akkermansia* and *Bifidobacterium*), and a unique fecal metabolome rich in pro-inflammatory metabolites.	Fujimura et al., 2016
3	Stokholm et al.	2018	Copenhagen	Cohort of 700 children recruited in pregnancy. During the first 5 years of life, children were prospectively followed up for deep clinical phenotyping at 11 scheduled visits, including 1 week, 1, 3, 6, 12, 18, 24, 30, and 36 months, and then once a year.	16S rRNA gene amplicon sequencing of the V4 region.	The immature microbial composition of 1-year-old children could increase the risk of developing asthma at the age of 5. It is only evident in children born to mothers with asthma, which indicates that the immaturation of gut microbiota in the first year of life is a crucial determinant of the elevated risk of asthma. Maternal asthma status doesn’t influence the composition and maturation of gut microbiota in early life. In the healthy adult human gut, the most abundant Firmicutes families are *Ruminococcaceae* (including *Faecalibacterium* and *Ruminococcus*) and *Lachnospiraceae* (including *Roseburia* and *Lachnospiraceae incertae sedis*). In children with later asthma, the relative abundance of the above genera is lower, which indicates an overall delay in microbial maturation.	Stokholm et al., 2018

In 2015, Arrieta et al. (2015) compares the gut microbiota of 319 infants and shows that infants at risk of asthma exhibit transient gut microbial dysbiosis during the first 100 days of life. The relative abundance of the bacterial genera *Rothia*, *Faecalibacterium*, *Veillonella*, and *Lachnospira* is significantly reduced in infants at risk of asthma. The decrease of bacterial taxa is accompanied by the dysregulation of enterohepatic metabolites and the decrease of fecal acetate levels. Inoculating these four bacterial taxa into germ-free mice can improve airway inflammation of their adult offspring, which proves the causal role of these four bacterial taxa in avoiding the development of asthma. Another important finding of this study is that infants with atopy (that is, allergy skin prick testing) and clinical wheeze data at 3 months of age have a significant decrease in fecal acetate. The conducted asthmatic animal models show that the butyrate, acetate, propionate, and SCFAs, which can stimulate dendritic cells and Tregs and prevent Th2-type immune responses (Holgate, 2012), have a protective effect on airway inflammation. In 2016, another study (Fujimura et al., 2016) indicated that neonatal gut microbiota dysbiosis led to CD4 + T cell dysfunction associated with childhood atopy, which affected the susceptibility of allergic asthma in children. These results proved that the first 100 days of human life represented a critical window of early life, in which dysbiosis of gut microbiotal was related to the risk of asthma and allergic disorders. In 2018, a study from Stokholm et al. (2018) demonstrated that the immature microbial composition of 1-year-old children could increase the risk of developing asthma at the age of 5. It is only evident in children born to mothers with asthma, which indicates that children born to mothers with asthma are vulnerable to gut microbiota. Meanwhile, maternal asthma status doesn’t influence the composition and maturation of gut microbiota in early life, which points to mechanisms other than strict genetic effects. The immaturation of gut microbiota in the first year of life is a crucial determinant of the elevated risk of asthma.

### Microbiota and Diabetes

Diabetes is a group of chronic metabolic diseases resulted from insulin secretion and (or) action deficiency. It is characterized by elevated blood glucose levels, which will seriously damage the eyes, heart, kidneys, nerves, and blood vessels over time (Al-Awar et al., 2016). According to etiological classification, type 1 diabetes (T1D) and type 2 diabetes (T2D) are the two most common types of diabetes. T1D accounts for about 5–10% of all types of diabetes, once known as insulin-dependent or juvenile diabetes. The pancreas of patients with T1D produces little or no insulin. The most common form is T2D, affecting 90 to 95% of patients with a family history of diabetes, which usually occurs in adults. T2D happens when the body is unable to produce enough insulin or resistant to insulin (Pascale et al., 2018).

T1D is an autoimmune disease. A meta-analysis based on 20 studies demonstrates that c-section delivery contributes a 20% increased risk of childhood-onset T1D, which may reflect differences in microbial exposure in early life (Cardwell et al., 2008). It has been shown that delivery mode has an important influence on the immunological function and the development of gut microbiota in infants (Huurre et al., 2008), both of which are related to the development of diabetes (Bonifacio et al., 2011). Leiva-Gea et al. (2018) have found that T1D is related to the differences in gut microbial composition. Compared with healthy children, the diversity of gut microbiota is significantly lower in patients with T1D. A case-control study (Murri et al., 2013), which conducted in 16 healthy children and 16 children with T1D, demonstrated that T1D was related to compositional changes in gut microbiota. It was found that compared with the healthy children, the bacterial number of Firmicutes and Actinobacteria decreased significantly while that of Bacteroidetes increased significantly in the children with T1D. And this change was associated with the glycemic level of the diabetic children. Moreover, the numbers of mucin-degrading bacteria, butyrate-producing bacteria and lactic acid-producing bacteria were significantly lower in the diabetic children than the healthy children, which were important to maintain gut integrity. The findings are helpful to develop strategies to control the development of patients with T1D by regulating their gut microbiota.

In 2020, Chavarro et al. (2020) suggested that c-section delivery was related to an increased risk of T2D of the offspring in adulthood. They conduct a prospective cohort study and find that, compared with vaginally delivered offspring, those delivered by c-section have a 46% higher risk of T2D in adulthood. It is known that delivery mode has an important influence on the gut microbiota of offspring (Dominguez-Bello et al., 2010, 2016; Shao et al., 2019). A study have showed that the gut microbiota is an environmental factor controlling energy metabolism, which is closely related to metabolic disorders such as T2D (Cani and Delzenne, 2007). It was reported that T2D was related to the dysbiosis of gut microbiota (Zhang et al., 2020) rather than the effect of a simple increase in diversity or a single microbe (Wu et al., 2010). The fecal samples of 16 patients with T2D and 12 healthy individuals were collected in this study and found that the fecal microbial composition in diabetic group was different from the healthy group. There are significant differences in the number of *Bifidobacterium* and the similitude of bacterial community between diabetes group and healthy group. It is reported that the genera of *Bifidobacterium*, *Bacteroides*, *Roseburia*, *Akkermansia*, and *Faecalibacterium* are negatively correlated with T2D, while the genera of *Blautia*, *Fusobacterium*, and *Ruminococcus* are positively correlated with T2D (Gurung et al., 2020).

### Microbiota and Obesity

In 2016, the World Health Organization (WHO) evaluated that 39% (39% of men and 40% of women) of adults older than 18 were overweight. Between 1975 and 2016, the global prevalence of obesity almost tripled. Obesity is a major risk factor of developing chronic diseases, which can lead to cardiovascular diseases (CVDs), type II diabetes (T2D), musculoskeletal disorders and cancers (breast, liver, gallbladder, kidney, colon, endometrial, ovarian, and prostate) (Li et al., 2017). An ongoing prospective cohort study, enrolls 116,671 women aged 24 to 44 years, conducted in 1989. Throughout the follow-up period, they find that being born by c-section is related to an 11% higher risk of obesity. There is growing evidence that obesity in adulthood among individuals born by c-section is associated with changes in gut microbiota (Cani and Delzenne, 2007). It was shown that mice in c-section group gain more body mass and the phenotype of females is even stronger after weaning (Martinez et al., 2017). Compared with the control mice, C-section-born mice lack the dynamic developmental changes of gut microbiota. Here, they found that the bacterial taxa which are related to vaginal delivery, such as *Clostridiales*, *Ruminococcaceae*, and *Bacteroides*, have previously been related to lean phenotypes in mice. The results prove that there is a causal relationship between c-section and weight gain, and could support that maternal vaginal bacteria participate in the normal metabolic development of offspring. The results also suggest that maternal vaginal microbiota is necessary for normal metabolic development during delivery and provide important new information in the context of the global obesity epidemics. Generally, the change of microbial composition may result in weight gain through three ways: (1) microbiota may modulate the expression of gut gene, resulting in the increases of adipose and free fatty acids levels; (2) microbiota can convert indigestible food into biochemical absorbable nutrients, which can increase energy harvest; and (3) gut microbiota induce an inflammatory state of obesity by activating lipopolysaccharides (Pei et al., 2014). Therefore, dysbiosis of gut microbiota induced by c-section would be a target for prevent obesity (Isolauri, 2017).

## Dysbiosis of the Gut Microbiota Induced by Cesarean Section Affects Activation of Intestinal Epithelial Cells and Immune System

### C-Section and Activation of Intestinal Epithelial Cells

Intestinal epithelial cells (IECs) are non-hematopoietic cells. They resist external antigens by forming a physical barrier (Goto, 2019) and are the first defensive line of mucosal surface barrier. The activity of IECs greatly influence the gut microenvironment and immunity (Adachi et al., 2016). Lotz et al. (2006) have found that vaginally born mice show spontaneous activation of IECs and tolerance to Lipopolysaccharide (LPS) within two hours after birth. LPS, a product of microorganisms and their cell wall, could stimulate the innate immune response of immature fetal enterocyte and increase the production of IL-8 by activating the transcription factor NF-κB (Fusunyan et al., 2001). These findings can be confirmed by the detection of IκB-α phosphorylation, nuclear translocation of the NF-κB subunit p65 and transcriptional activation of chemokine MIP-2. However, this phenomenon is not found in c-section delivered neonates or in TLR4-deficient mice. The loss of LPS responsiveness after birth is related to the down-regulation of IL-1 receptor-associated kinase 1 after transcription, which plays a significant role in epithelial TLR4 signaling *in vitro*. The decreasing levels of TLR4 combined with the increasing levels of IκB-α expression by IECs effectively increases the threshold of immune activation in intestinal epithelium (Francino, 2018). IECs acquire TLR tolerance directly by exposure to exogenous endotoxin immediately after birth, which can promote subsequent birth colonization and maintain a stable intestinal host-microbiota homeostasis. However, intestinal epithelium cells of c-section infants could not activate of immediately after birth by LPS and then result in dysbiosis of microbiota.

### C-Section and Development of Immune System

Delivery mode strongly affect the early neonatal microbial exposure and immune environment. Labor can induce immune responses in the uterine cavity. These intrauterine immune responses will not occur in elective c-sections, which can affect the immune environment of neonate (Thornton et al., 2003). The first few months of life are a critical time window in the establishment of tolerance and the development of the immune system, delivered by c-section will affect the lifelong risk of developing immune diseases (Francino, 2018).

Delivery mode could make a difference in the frequencies of lymphocyte subset in full-term newborns (Pittard et al., 1989). The characteristics of full-term c-section neonates are that T cells and helper T cells increase significantly, while natural killer cells decrease significantly. In contrast, the characteristics of vaginally delivered neonates are that the frequency of T cells and helper T cells decrease significantly, while natural killer cells increase significantly (Samelson et al., 1992; Cuppari et al., 2015). To a large extent, immune tolerance to environmental antigens is mediated by peripheral Foxp3 + regulatory T cells (Knoop et al., 2017). It was found that the adult mice delivered by c-section had lower proportions of tolerogenic CD103 + dendritic cells and Foxp3 + regulatory T cells (Hanse et al., 2014; Zachariassen et al., 2019). In contrast, the Foxp3 + regulatory T cells of vaginally delivered neonates are elevated (Yildiran et al., 2011). T and B lymphocyte subpopulations of c-section delivered infants are increased. The positive rate of cell surface markers of B lymphocyte subpopulations is obviously higher in infants delivered by elective c-section (Gasparoni et al., 1992).

**FIGURE 1:**
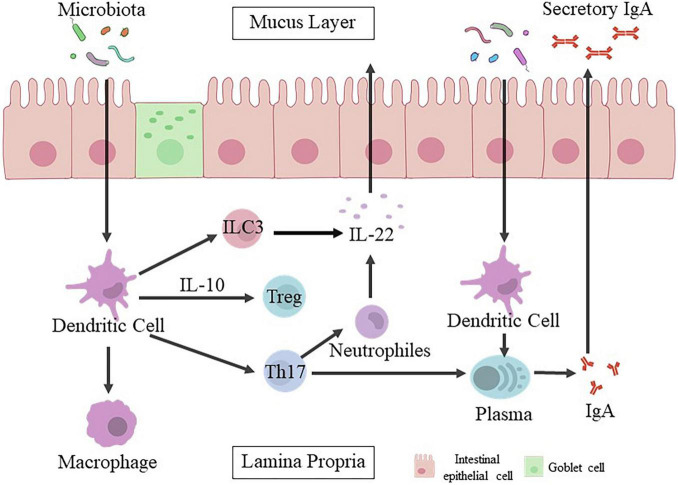
Intestinal epithelial cells mediate the crosstalk between gut microbiota and host immune responses. The mucosal barrier formed by intestinal epithelial cells can prevent the conflict between gut microbiota and host immune cells, and keep bacteria away from the epithelial surface. Microbial signals are captured by epithelial cells through their specific receptors directly or transmitted by dendritic cells (DCs) to activate other innate immune cells, including innate lymphocytes (ILC3) and macrophages (Mφ). Dendritic cells activated by bacteria are also involved in the recruitment and activation of adaptive immune cells. Plasma cells release IgA. Once IgA is attached to the polymeric immunoglobulin receptor (pIgR), it will enter the gut lumen in the form of secretory IgA (sIgA). Besides, various populations of CD4 + T cells are induced simultaneously, and their function is balanced to maintain physiological inflammation, especially under the control of Treg and IL-10. Th17 production (T helper 17).

Changing bacterial colonization at the intestinal level by c-section might play a important role in affecting the development of immune system (Cho and Norman, 2013). Gut microbiota is closely related to immunity system (Figure 1), which could promote the development of mesenteric lymph nodes, isolated lymphoid follicles, gut-associated lymphoid tissue Peyer’s patches and/or recruitment of mature immune cells (Renz et al., 2011; Olin et al., 2018). Abnormal intestinal colonization in c-section delivered infants may prolong postnatal immunological immaturity, and hinder proper immune initiation, leading to increase the risk of later immune diseases (Ly et al., 2006). Colonization of gut microbiota in neonates initiates the immune system and lead to the imbalance of Th1/Th2.56 The levels of Th1 related chemokines CXCL10 and CXCL11 are significantly lower in the blood of c-section delivered infants. C-section is associated with delayed colonization of the Bacteroidetes phylum, reduced diversity of total microbiota and decreased Th1 responses in the first two years after birth (Jakobsson et al., 2013).

Cytokines are of great importance in labor, which can affect the neonatal immunity (Yektaei-Kari et al., 2007). Vaginal delivery can promote the production of cytokines. However, cord blood from c-section delivered infants with decreased levels of IFN-γ and TNF-α can damage their production (Keelan et al., 2003). A prospective study find that the levels of IL-6, IL-1β, IFN-γ, and TNF-α in c-section delivered neonates are significantly lower than those in vaginally delivered neonates (Malamitsi-Puchner et al., 2005). Puff et al. (2015) have found that compared with vaginally delivered infants, the concentrations of cytokines IL-8 and IFN-γ is lower in c-section delivered infants. This study also shows that c-section delivered infants have lower cytokine release in early childhood than vaginally delivered infants. Besides, the level of immunoglobulin (Ig) in c-section delivered infants is lower than that in normal vaginal delivery. Bonifacio et al. (2011) devise a substantial cohort of young infants and find that compared with vaginally delivered infants, 1-year-old infants delivered by c-section contain more immune cells secreting IgA and IgG. The cord blood level of IgG in c-section delivered infants is significantly lower than that in normal vaginally delivered infants (Agrawal et al., 1996). However, the cord blood level of IgE in c-section delivered infants is higher than that in vaginally delivered infants (Petrovičová et al., 2016).

## Probiotics May Prevent and Treat Cesarean Section Related Gut Diseases

Probiotics are live micro-organisms which play a healthy role beyond the inherent general nutrition in the host after adequate intake. Probiotics could improve the dysbiosis of gut microbiota and restore gut function (Li et al., 2020). *Lactobacillus* and *Bifidobacterium* are dominant in maternal birth canal and also belong to probiotics (Yang et al., 2019). The undesired alterations in the composition and function of microbiota caused by caesarean section can be corrected by supplementing a probiotics mixture to infants (Korpela et al., 2018), which may be associated with the prevention and treatment of c-section related intestinal diseases.

Food allergy results from the deficiency of immune tolerance mechanism, which is regulated by the composition and function of gut microbiota. Selected probiotic strains could play the role of immune tolerance mechanisms (Paparo et al., 2019), and as a method of prevention and treatment of food allergy. The reintroduction of certain commensal microbes, such as Clostridia, could prevent or alleviate allergic symptoms in clinical trials and animal models (Shu et al., 2019). Besides, oral probiotics represent a possible treatment for asthma and allergic diseases (Kang et al., 2017). Studies show that probiotic supplementation can treat acute gastroenteritis in children and decrease the risk of developing necrotizing enterocolitis in premature infants (Bertelsen et al., 2016). Stewart et al. (2018) find that for people with potential T1D, early probiotic supplementation (within 27 days after birth) can reduce the risk of islet autoimmunity when compared with no probiotic supplementation or probiotic supplementation after 27 days. Minami et al. (2018) establish a randomized, double-blind, placebo-controlled trial and demonstrate that probiotic strain *B. breve* B-3 has the potential to reduce body fat in healthy pre-obese individuals. This probiotic strain can be used to prevent the accumulation of body fat and related metabolic disorders in pre-obese individuals. Moreover, although the composition of *in situ* microbiota remains unchanged, supplementation of *B. lactis* NCC2818 can further improve intestinal barrier function, immunity, host metabolism and host-microbiota co-metabolism (Lewis et al., 2017).

## Conclusion

Delivery mode has impacts on the establishment and development of gut microbiota in infants. C-section could lead to dysbiosis of gut microbiota. Cesarean section affects the activation of IECs and the development of immune system through a variety of mechanisms. Long-term dysbiosis of gut microbiota will lead to some c-section related autoimmune and metabolic disorders. Probiotics can improve the dysbiosis of gut microbiota and restore gut function, which may be related to the prevention and treatment of c-section related intestinal diseases.

There are some questions that can be used in clinical research in the future:

1. There are few studies on the mechanisms of c-section induced alterations of immune system in offspring. What are the specific differences and mechanisms between the specific immune system of c-section and vaginal delivered infants? Further study is needed.
2. It is known that *L. acidophilus* and *Bifidobacterium longum* can upregulate the levels of SERT mRNA and protein in intestinal epithelial cells. Whether the supernatants of these two species can also improve gastrointestinal sensation and intestinal motility by regulating the expression of SERT?
3. Could probiotics activate the IECs to enable neonate delivered by c-section to acquire the same immune tolerance as vaginal delivered neonate?

## Author Contributions

CZ, LL, and ZL contributed to conception and design of the work. BJ and XX contributed to the acquisition, analysis, and interpretation of data for the manuscript. CZ and LL drafted the manuscript. XZ and YL critically revised and reviewed the manuscript for important intellectual content. All authors contributed to the article and approved the submitted version.

## Conflict of Interest

The authors declare that the research was conducted in the absence of any commercial or financial relationships that could be construed as a potential conflict of interest.

## Publisher’s Note

All claims expressed in this article are solely those of the authors and do not necessarily represent those of their affiliated organizations, or those of the publisher, the editors and the reviewers. Any product that may be evaluated in this article, or claim that may be made by its manufacturer, is not guaranteed or endorsed by the publisher.
